# ATTR-FAP: liver transplantation vs oral medication, how and when

**DOI:** 10.1186/1750-1172-10-S1-I8

**Published:** 2015-11-02

**Authors:** Bo Goran Erikzon

**Affiliations:** 1 Center for Surgical Sciences, Karolinska Institutet, Karolinska University Hospital Huddinge, 141 86 Stockholm, Sweden

## Background

Liver transplantation (LTx), introduced in 1990, has served as the only available treatment with capacity to halt the progress of disease in transthyretin amyloidosis. For the most common variant, Val30Met, the effect of a new liver is well known leading to stabilization in the majority of patients. However, not all patients are helped by transplantation. Progress of cardiac amyloidosis is not uncommon necessitating both liver and heart transplantation (LTx/HTx). The effect of LTx is less well studied in patients with non-Val30Met mutations, and outcome has generally been inferior to that seen in patients with the Val30Met mutation. Large variations in survival, not only between different mutations but also between mutations with similar phenotypes, have been noted and it is clear that each mutation needs to be considered individually. Some mutations have similar long-term survival as the Val30Met, while LTx is not to be recommended for other mutations. Several novel pharmaco-therapeutical approaches have emerged over the last years and may provide a more attractive and less invasive treatment for this patient population.

## Methods

Data concerning outcome after LTx for ATTR amyloidosis was extracted from the FAPWTR registry. Survival rates were analyzed by the Kaplan-Meier method and Log-Rank test.

## Results

In total, 58 different mutations were treated by LTx alone or by LTX/HTx. Data from more than 2000 patients were accumulated from 77 collaborating liver transplant centers. Overall, 20-year survival after LTx was 55.3%. Modified Body Mass Index, early onset of the disease, disease duration before transplantation and Val30Met versus non Val30Met mutations were independent significant survival factors. Cardiovascular death was markedly more common than that observed in patients undergoing LTx for end stage liver disease. There has been a significant drop in the annual number of transplants over the last years following the introduction of pharmacotherapy in Europe. A careful evaluation regarding the effect of new promising pharmacotherapies in relation to LTx is of the outmost importance. There is a risk, that patients not responding to pharmacotherapy may be exposed to a less favorable surgical outcome because LTx is delayed. Furthermore, it is unknown if patients not responding to LTx will respond better to alternative treatment and vice versa.

## Conclusion

Long-term survival after LTx for many TTR variants is excellent. Several new promising pharmacological treatments are under evaluation. In order to determine the most optimal use, these pharmaco-therapeutical approaches must be compared to the existing surgical therapy. Perhaps the best treatment for some patients will be a combination of pharmacotherapy and surgery.

